# It’s good to know what to BACE the specificity of your inhibitors on

**DOI:** 10.1172/JCI183677

**Published:** 2024-08-15

**Authors:** Aoife Murray, Ana Muñiz-García, Ivan Alić, Dean Nižetić

**Affiliations:** 1 The Blizard Institute, Faculty of Medicine and Dentistry, Queen Mary University of London, London, United Kingdom.; 2 Department of Anatomy, Histology and Embryology, Faculty of Veterinary Medicine, University of Zagreb, Zagreb, Croatia.

## Abstract

Production, aggregation, and clearance of the amyloid β peptide (Aβ) are important processes governing the initial pathogenesis of Alzheimer’s disease (AD). Inhibition of β-site amyloid precursor protein (APP) cleaving enzyme (BACE1) (one of two key proteases responsible for Aβ production) as an AD-therapeutic approach so far has failed to yield a successful drug. BACE1 and its homologue BACE2 are frequently inhibited by the same inhibitors. Several genetic and cerebral organoid modeling studies suggest that BACE2 has dose-dependent AD-suppressing activity, which makes its unwanted inhibition potentially counterproductive for AD treatment. The in vivo effects of an unwanted cross inhibition of BACE2 have so far been impossible to monitor because of the lack of an easily accessible pharmacodynamic marker specific for BACE2 cleavage. In this issue of the *JCI*, work led by Stefan F. Lichtenthaler identifies soluble VEGFR3 (sVEGFR3) as a pharmacodynamic plasma marker for BACE2 activity not shared with BACE1.

## BACE1 and BACE2 in Alzheimer’s disease

**Figure 1:**
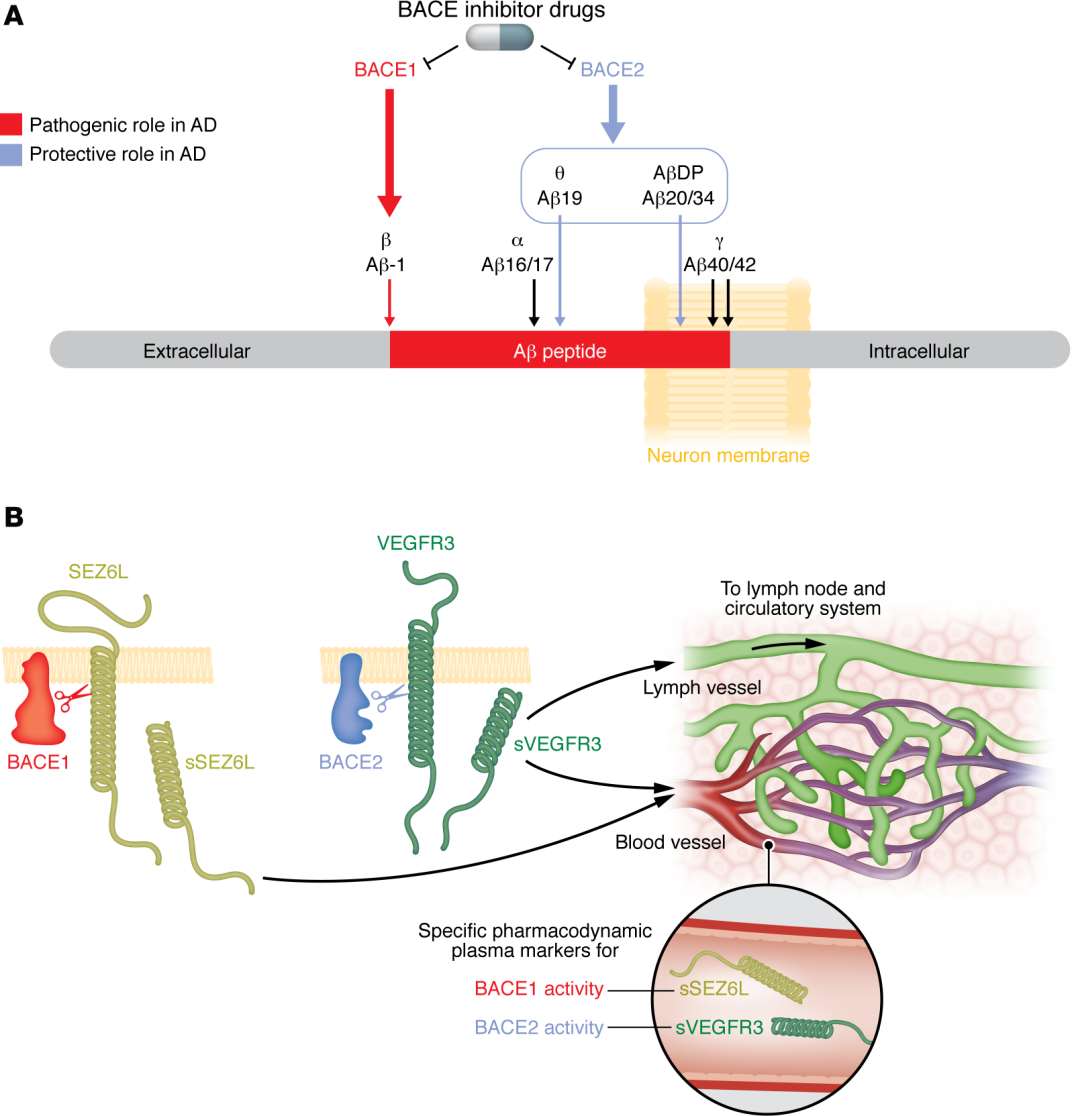
sSEZ6L and sVEGFR3 provide pharmacodynamic markers specific to BACE1 and BACE2 cleavage activities. (**A**) BACE1 and BACE2 predominantly cleave APP or Aβ at specific sites. BACE inhibitor drugs block activity of both proteases. (**B**) BACE1 cleavage of SEZ6L releases sSEZ6L into the plasma, while BACE2 cleavage of VEGFR3 releases sVEGFR3 into the plasma and lymphatic vessels. Serum levels of sSEZ6L and sVEGFR3 specifically reflect BACE1 and BACE2 activity, respectively.

A recent breakthrough in the treatment of the initial stages of Alzheimer’s disease (AD) is marked by the first approved disease-modifying drugs, which are all monoclonal antibodies recognizing aggregates of the amyloid β peptide (Aβ) (1). The approval vindicates the importance of Aβ as a therapeutic target for AD, with the production, aggregation, and clearance of Aβ as strategic focal points (1). However, whereas an increased production of Aβ clearly causes early onset AD (2), chemical inhibition of the proteases (i.e., β-secretase and γ-secretase) responsible for Aβ production have so far failed to yield a successful drug, despite extensive efforts by multiple pharmaceutical industries (3). Aβ peptide is carved out of the amyloid precursor protein (APP) through sequential cleavage by β-secretase and γ-secretase (Figure 1A). β-Site APP cleaving enzyme (BACE1) has been identified as the main enzyme performing the critical amyloidogenic β-secretase cut in human and rodent brain (4), while the role of its homologue BACE2 remains poorly understood. BACE1 and BACE2 are both type I transmembrane aspartyl proteases, have a similar length, and share 59% identify in the amino acid sequence (5), which is the reason why chemical inhibitors designed to inhibit BACE1 in most cases also inhibit BACE2, to a varying extent (3). Early studies in mouse brain established BACE1 as having strong expression in mouse neurons, with the expression of BACE2 in the brain remaining very low (6). BACE2-knockout mice (unlike those with BACE1 deficiency) did not show decreased amyloid plaque load in AD models (7). These findings resulted in a relative neglect of BACE2 as a role player in AD and rendered the cross inhibition of BACE2 by the BACE1 inhibitors less important for AD treatment. However, multiple studies have since shown that human neurons express much more BACE2 than mouse neurons (8, 9). Studies in a variety of human cell-line models overexpressing or silencing BACE2 revealed a reproducible effect that dose of BACE2 has on the level of secreted Aβ: *BACE2* overexpression decreases secreted Aβ levels (10–14), while siRNA silencing of *BACE2* increases Aβ levels (15). However, the in vivo effects of an unwanted cross inhibition for BACE2 were difficult to prove and impossible to monitor because of the lack of an easily accessible pharmacodynamic target of BACE2 cleavage. Unlike BACE1, whose cleavage of APP and SEZ6L could be pharmacodynamically measured in cerebrospinal fluid (CSF) and partly observed in blood (16, 17), the only verified in vivo targets of BACE2 until now remained TMEM27 and PMEL, whose cleavages by BACE2 altered glucose homeostasis in β-islet cells and pigmentation in melanocytes, respectively. The manuscript by Schmidt et al., a multidisciplinary work led by Lichtenthaler and published in this issue of the *JCI* (18), identifies an easily accessible pharmacodynamic marker for BACE2 activity (not shared with BACE1) in human, nonhuman primate, and rodent plasma samples.

## sVEGFR3 as a plasma marker specific for BACE2 activity

Schmidt et al. (18) took advantage of the mouse models in which either or both *Bace1* or *Bace2* genes were knocked out. The authors first compared the plasma proteomes of these mouse models and found one protein, VEGFR3, strongly (approximately 8-fold) reduced only in models where *Bace2* (and not *Bace1*) was knocked out. They verified this finding using a Meso Scale Diagnostics immunoassay (MSD-assay) and immunoblot assays and by independently knocking down *BACE2* in human cells. All peptides detecting the VEGF3R mapped to its N-terminal ectodomain, suggesting that BACE2 might be performing an in-membrane proteolytic cleavage of VEGFR3, releasing its ectodomain. Subsequent experiments confirmed that the shedding of the ectodomain (soluble VEGFR3 [sVEGFR3]) occurred by the action of BACE2 and identified the BACE2-cleavage domain within VEGFR3. The authors provided further evidence for physiological cleavage of VEGR3 by BACE2 within primary human lymphatic endothelial cells. They also used a zebrafish model to confirm that BACE2 modulates VEGFR3 signaling in vivo during lymphangiogenesis (18).

Most importantly, Schmidt et al. showed that sVEGFR3 levels in plasma of mice and nonhuman primates responded rapidly to decreased levels of BACE2 activity, and this effect was also present in *Bace1*-knockout, but not in *Bace2*-knockout, mice (18). The same experimental system also implicated BACE1 (and not BACE2) activity in the regulation of plasma levels of sSez6L. Thus, the authors posited that plasma levels of sVEGFR3 and sSEZ6L could serve as in vivo pharmacodynamic markers specific to BACE2 and BACE1 cleavage activities, respectively (Figure 1B). Notably, two BACE1 inhibitors, which underwent clinical trials for AD, also cross inhibited BACE2’s ability to cleave VEGFR3, and the effect was reflected by plasma levels of sVEGFR3 in patients with AD who were treated with those inhibitors (18).

## BACE2 activity–specific biomarker is strategically important for AD

Seemingly paradoxical sets of data require clarification in the field: on one hand, BACE2 has very little to do with proamyloidogenic β-secretase cleavage producing Aβ and BACE1 has everything to do with this activity (4, 7). On the other hand, two separate studies found SNPs around the *BACE2* locus were associated with increased risk of late-onset sporadic AD and were associated with the levels of brain Aβ load or Aβ1-42 in the CSF of the same cohorts (12, 19). No such associations were ever reported for SNPs around the *BACE1* locus. The simplest explanation involves BACE2 in the prevention or clearance of Aβ and/or its aggregates. Several lines of evidence converge in support of this hypothesis. First, studies using engineered human cell lines and synthetic Aβ peptides have shown that, besides cleavage at the β-secretase site of APP (before amino acid 1 of the Aβ sequence), BACE2 is also capable of cleaving APP fragments after amino acid 19 of the Aβ sequence (so-called φ-secretase activity) (11, 14) and degrading Aβ by cleaving at the point after amino acids 20 and 34 of the Aβ sequence (known as Aβ-degrading protease [AβDP] activity) (10, 11, 13) (Figure 1A). Both of these activities are in theory protective against AD pathogenesis, as they prevent and degrade Aβ. Second, a recent study (20) has shown that an increased presence of the cleavage products of all of these theoretical cuts is measurable in the CSF of individuals who have a genetically constitutionally increased dose of BACE2, those with Down syndrome (DS), as this syndrome is caused by aneuploidy, with an extra chromosome 21 that harbors *BACE2*. The profile of Aβ proteolytic fragments from the CSF of individuals with DS was also similar to that from cerebral organoids derived from DS-induced pluripotent stem cells (iPSCs), and this was shown to be caused by the triplication of *BACE2* and not of *APP* (20). Third, artificial elimination of one copy of *BACE2* by CRISPR/Cas9 editing in the trisomy-21 iPSCs caused an extremely accelerated presence of a triad of AD-like neuropathological signs: amyloid plaque–like deposits, pathologically conformed intraneuronal tau, and progressive neuronal loss (20). A very similar result was reproduced in another study of cerebral organoids from a patient with Hirschsprung’s disease bearing a mutation causing a BACE2 haploinsufficiency (21). These studies put together suggest that a disbalance of gene doses of *APP* and *BACE2* (3:2 or 2:1) accelerate AD-like pathology in cerebral organoids consisting mainly of cortical neurons (20, 21). The findings also imply that BACE2 is a dose-dependent AD suppressor (20). Importantly, the Aβ-degrading activity of BACE2 was cross inhibited by BACE1 inhibitors that underwent clinical trials in a dose-dependent fashion (20), emphasizing once more the paramount importance of determining the specificity of action for BACE inhibitors. The BACE inhibitor specificity has hitherto been nearly impossible to establish in vivo in human patients. sVEGFR3 as a pharmacodynamic marker specific for the BACE2 activity (18) allows for assessment of unwanted cross inhibition of BACE2.

## Remaining open questions

A perfectly logical question persists: if a potent BACE1-inhibitor drug substantially lowers Aβ levels in the plasma, brain, and CSF (16), why does it matter if the same inhibitor also cross inhibits the Aβ-degrading activity of BACE2? Several indirect lines of evidence provide reasons why, potentially, it could matter. While strong inhibition of production of new Aβ may prevent the primary aggregation of Aβ by reducing the concentration of the newly produced Aβ monomers, the brains of patients undergoing this therapy are full of amyloid plaques. Such plaques are an abundant source of soluble oligomerizing Aβ units released from the fibrils and/or generated by secondary nucleation on the fibrils, which are thought to be particularly toxic to neurons (22). The fact that amyloid PET–positive plaque load was reduced in the brains of patients that underwent BACE1-inhibitor treatment (23) proves that the brain possesses powerful mechanisms of eliminating the Aβ released back into the soluble pool from the existing fibrils. While it has yet to be established, BACE2 could serve as a contributing factor in this elimination machinery, especially considering the presence of the Aβ1-34 peptide, which is a product of BACE2 activity, as AβDP, was seen accumulating strongly in neurons near the plaques (20, 24). It remains to be determined to what extent the unwanted cross inhibition of BACE2 cleavage of VEGFR3 also reflects the inhibition of its Aβ-degrading activity.

Lichtenthaler and colleagues also suggest that their assay for the detection of sVEGFR3 in plasma should be further improved by the development of neoepitope-specific antibodies that would not detect the sVEGFR3 product of alternative splicing, but be completely specific for the shed sVEGFR3 generated by the BACE2 cut (18). Once this is achieved, it would be really interesting to examine the association of sVEGFR3 plasma levels with overall risk, severity, and age of onset of AD.

## Version 1 08/15/2024

Electronic publication
